# Constructing Amorphous‐Crystalline Interfacial Bifunctional Site Island‐Sea Synergy by Morphology Engineering Boosts Alkaline Seawater Hydrogen Evolution

**DOI:** 10.1002/advs.202309927

**Published:** 2024-03-18

**Authors:** Pengliang Sun, Xiong Zheng, Anran Chen, Guanghong Zheng, Yang Wu, Min Long, Qingran Zhang, Yinguang Chen

**Affiliations:** ^1^ State Key Laboratory of Pollution Control and Resource Reuse School of Environmental Science and Engineering Tongji University Shanghai 200092 P. R. China; ^2^ Shanghai Institute of Pollution Control and Ecological Security Shanghai 200092 P. R. China; ^3^ School of Materials and Energy Yunnan University Kunming 650091 P. R. China

**Keywords:** amorphous‐crystalline heterostructures, hierarchical structure, hydrogen evolution reaction, nanocages‐nanoclusters, seawater electrolysis

## Abstract

The development of efficient and durable non‐precious hydrogen evolution reaction (HER) catalysts for scaling up alkaline water/seawater electrolysis is highly desirable but challenging. Amorphous‐crystalline (A‐C) heterostructures have garnered attention due to their unusual atomic arrangements at hetero‐interfaces, highly exposed active sites, and excellent stability. Here, a heterogeneous synthesis strategy for constructing A‐C non‐homogeneous interfacial centers of electrocatalysts on nanocages is presented. Isolated PdCo clusters on nanoscale islands in conjunction with Co_3_S_4_ A‐C, functioning as a bifunctional site “island‐sea” synergy, enable the dynamic confinement design of metal active atoms, resulting in excellent HER catalytic activity and durability. The hierarchical structure of hollow porous nanocages and nanoclusters, along with their large surface area and multi‐dimensional A‐C boundaries and defects, provides the catalyst with abundant active centers. Theoretical calculations demonstrate that the combination of PdCo and Co_3_S_4_ regulates the redistribution of interface electrons effectively, promoting the sluggish water‐dissociation kinetics at the cluster Co sites. Additionally, PdCo‐Co_3_S_4_ heterostructure nanocages exhibit outstanding HER activity in alkaline seawater and long‐term stability for 100 h, which can be powered by commercial silicon solar cells. This finding significantly advances the development of alkaline seawater electrolysis for large‐scale hydrogen production.

## Graphical abstract

Isolated PdCo clusters on nanoscale islands in conjunction with Co_3_S_4_ amorphous‐crystalline, functioning as a bifunctional site “island‐sea” synergy, enabling the dynamic confinement design of metal active atoms, resulting in excellent HER catalytic activity and durability.

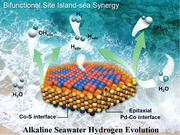

## 1 Introduction

The energy crisis and environmental challenges rooted in the widespread use of fossil fuels compel us to explore sustainable and clean energy.^[^ ^1^ ^]^ As an ideal clean fuel and energy carrier, hydrogen has recently sparked a new wave of research due to its exceptionally high energy density and zero carbon emissions.^[^ ^2^ ^]^ Electrochemical water splitting has emerged as the most promising method for high‐purity hydrogen production, given its relatively high energy exchange efficiency and absence of greenhouse gas emissions.^[^ ^3^ ^]^ As freshwater resources become increasingly scarce, the utilization of seawater or brine electrolytes for hydrogen production is of paramount importance.^[^ ^4^ ^]^ Currently, platinum (Pt) is extensively studied as the most effective catalyst for hydrogen production.^[^ ^5^ ^]^ However, the scarcity and high cost of Pt hinder its feasibility for large‐scale commercial applications in the field of hydrogen production.^[^ ^6^ ^]^ Therefore, the quest for low‐cost, highly stable, and active non‐platinum catalysts for stable hydrogen production in alkaline seawater remains a prominent focus and challenge.

Transition metal chalcogenides (TMCs) have gained significant attention due to their excellent intrinsic activity and the ability to fine‐tune their structures and compositions through nanoscale engineering.^[^ ^7^ ^]^ Among these TMCs, such as MoS_2_,^[^ ^8^ ^]^ Co_3_S_4_,^[^ ^9^ ^]^ and Ni_3_S_2_^[^ ^7^, ^10^ ^]^ have been extensively studied as electrocatalysts for various applications, particularly in electrochemical water splitting. Particularly, the construction of shell‐in‐hollow structures with diverse beneficial constituents and features can harness the internal voids of the hollow architecture, exposing abundant active sites, increasing the catalyst‐electrolyte interface contact area, and reducing mass/charge transport distances, which offers a promising avenue for accelerating the kinetics of hydrogen evolution reaction (HER).^[^ ^11^ ^]^ Beyond morphological engineering, atomic‐level heterostructure engineering offers an alternative avenue. When two distinct components come into contact to form a heterostructure, spontaneous atomic configuration, and electronic structure reorganization occur in the vicinity of the heterointerface.^[^ ^12^ ^]^ Through the construction of coupled interfaces and the synergistic effects of heterostructures, electron transfer, active site modulation, and catalytic activity can be effectively tuned.^[^ ^13^ ^]^ However, the HER performance of TMCs is constrained by several issues, such as poor charge transfer, low reactivity of active site, insufficient electrical contact with supported catalysts, and limited electron transfer efficiency.^[^ ^7b^ ^]^ In particular, the formation of S‐H_ads_ bonds on the chalcogenide surface, while favorable for hydrogen atom adsorption, is unfavorable for the conversion of H_ads_ to H_2_.^[^ ^7a^ ^]^ Therefore, there is an urgent need to enhance the HER performance of TMCs through comprehensive control of their structural morphology and heterostructure engineering. Rational design of heterostructures, active sites, optimization of energy adsorption, and acceleration of H_2_O dissociation kinetics are essential for achieving large‐scale electrolysis.

Interface engineering is widely employed to enhance catalytic activity by effectively regulating the electronic structure and surface properties of metal chalcogenides.^[^ ^14^ ^]^ Among them, the amorphous‐crystalline (A‐C) heterostructure is recognized as a promising electrocatalyst.^[^ ^15^ ^]^ These materials harness the combined advantages of amorphous and crystalline structures and exhibit unconventional atomic arrangements at heterostructure interfaces.^[^ ^16^ ^]^ In comparison to crystalline materials, amorphous materials exhibit superior electrocatalytic HER performance due to their abundant active sites, defects, and unsaturated electron configurations, which attributes arise from the disordered atomic structure inherent to amorphous materials.^[^ ^17^ ^]^ Jin et al.^[^ ^15b^ ^]^ achieved excellent electrocatalytic performance through phase structure engineering in the optimized Ni‐TPA@NiSe/NF heterostructure. Furthermore, the geometric mismatch in the heterointerface region may induce localized lattice strain, consequently altering defect formation energies and migration barriers, leading to the generation of a substantial number of lattice defects. Yang et al.^[^ ^18^ ^]^ emphasized the coupling of oxygen vacancies with interface engineering, introducing a novel amorphous/crystalline CrO_x_‐Ni_3_N heterostructure, where high‐energy lattice defects were confirmed to be favorable active centers for electrochemical reactions. However, due to the demands of practical applications and the requirements of cutting‐edge technologies, the development of heterogeneous catalysts that process both high HER catalytic activity, cost‐effectiveness, and the capability to achieve high stability in alkaline seawater electrolysis systems remains a significant challenge.^[^ ^17a^ ^]^ Therefore, it is of paramount importance to establish a simple and versatile method for the fabrication of electrocatalysts with high‐density A‐C interfaces.

Herein, we present a nanoscale island confinement strategy through in situ ion‐exchange‐induced amorphous‐crystalline heterostructure remodeling, to construct atomic‐layer seawater splitting catalysts with high activity and stability. The intricately designed 3D PdCo‐Co_3_S_4_ nanocages boast a well‐defined hierarchical nanocages‐nanoclusters structure, a high‐stability A‐C heterointerface, and accelerated charge/mass transfer capability, exhibiting significantly enhanced HER activity. Benefiting from the abundant morphological structure and numerous A‐C heterointerfaces, the PdCo‐Co_3_S_4_ heterostructure nanocages show a low overpotential of 98 mV to drive −10 mA cm^−2^ and long‐term stability, demonstrating superior performance compared to traditional cobalt sulfide‐based HER catalysts. Moreover, we have constructed an alkaline seawater electrolyzer system using solar photovoltaic panels, demonstrating excellent HER activity and long‐term stability over 100 h. This work not only paves the way for the synthesis of functional catalysts rich in crystalline and amorphous interfaces, but also offers novel insights into the design of efficient and stable catalysts for alkaline seawater electrolysis.

## 2 Results and Discussion

### 2.1 Synthesis and Structural Characterization

**Figure 1:**
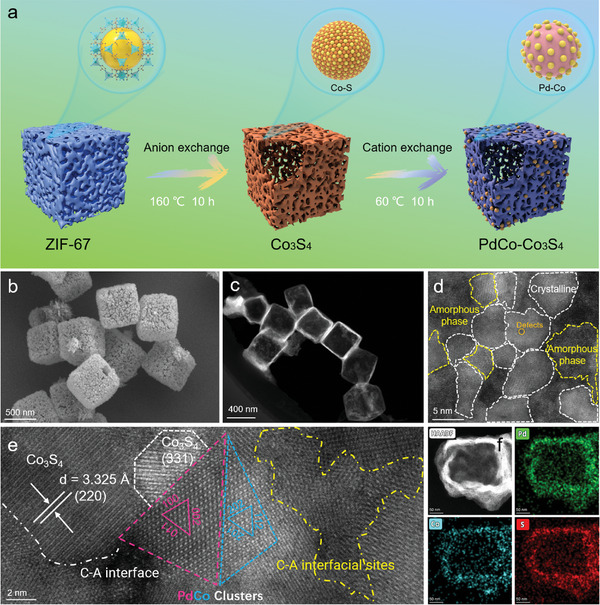
Structural characterization of the electrocatalysts. a) Schematic of the synthesis procedure of PdCo‐Co_3_S_4_. b) SEM and c) STEM images of PdCo‐Co_3_S_4_. d,e) HAADF‐STEM image of the amorphous‐crystalline heterostructures PdCo clusters‐Co_3_S_4_ nanocages. f) HAADF image and energy‐dispersive spectroscopy (EDS) element mappings of Pd, Co, and S for the amorphous PdCo‐Co_3_S_4_ sample.

According to the schematic diagram in **Figure** **1**a, uniform hollow Co_3_S_4_ nanoboxes were first prepared for the synthesis of porous PdCo‐Co_3_S_4_ nanocages using a cubic nanocrystal ZIF‐67 as the initial precursor via an improved Kirkendall method.^[^ ^9a^ ^]^ The cation exchange reaction between Co^2+^ and Pd^2+^ was achieved through a solvothermal reaction. Due to differences in their solubility product constants (k_sp_), a natural non‐equivalent cation exchange reaction occurred between Co^2+^ and Pd^2+^. However, this led to changes in the coordination modes due to chemical valence mismatch, resulting in a porous morphology enriched with defects. Due to the reducing property of L‐ascorbic acid, a small amount of free Pd/Co ions will be reduced to monometallic, thus obtaining a small amount of PdCo clusters. The presence of numerous defects and surface PdCo clusters enhanced the efficiency of electron transfer from the catalyst to protons, thereby achieving efficient HER.

**Figure 2:**
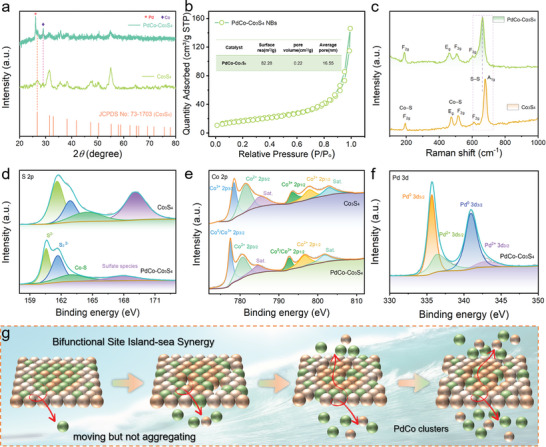
Spectroscopy characterization and chemical states of PdCo‐Co3S4. a) XRD patterns of PdCo‐Co_3_S_4_ nanocages along with the standard PDF cards for Co_3_S_4_. b) Nitrogen adsorption–desorption isotherms and (inset) pore‐size distribution and c) Raman spectra of the PdCo‐Co_3_S_4_. High‐resolution XPS spectra of d) S 2p, e) Co 2p, and f) Pd 3d for the as‐prepared catalysts. g) Structural schematic diagram.

In particular, the images captured by field emission scanning electron microscopy (FE‐SEM) (Figure 1b) and high‐angle annular dark‐field scanning transmission electron microscopy (HAADF‐STEM) (Figure 1c) reveal the distinctive hollow nano‐cubic structure of PdCo‐Co_3_S_4_. Within the STEM image (Figure 1d), a nanoscale island‐like arrangement of PdCo clusters in conjunction with Co_3_S_4_ nanocages is evident, showcasing the “moving but not aggregating” design of metal active sites (**Figure** **2**g). The strategy of dual‐functional site synergy in the “ island‐sea synergy” architecture. The strategy involves loading PdCo nanoclusters onto Co_3_S_4_ nano‐islands and dispersing PdCo nanoclusters, enhancing the beneficial catalytic effect of dispersed PdCo atoms. PdCo atoms remain dispersed in a reducing environment, that is, “mobile but not aggregated”, Abundant A‐C heterointerfaces and the synergistic effect of nanoclusters significantly improve HER activity. Consequently, this unique multi‐tiered architecture facilitates rapid electron and ion transfer. Furthermore, notable atomic defects and lattice distortions are discernible, and the lattice distortion serves to lower energy barriers and augment active edge sites, thus enhancing the electrochemical performance. Moreover, through HAADF‐STEM observations (Figure 2e), it was possible to further confirm lattice fringes with spacings of 0.33 and 0.21 nm, which could be attributed to the (220) and (311) crystal planes of Co_3_S_4_ (JCPDS No.73‐1703), respectively. Furthermore, within the regions demarcated by white dashed lines, an abundance of interfaces was evident, encompassing amorphous, low‐crystalline, and well‐crystallized domains. It was also observed that Pd and Co clusters coexisted at heterojunctions, which highly discrete clusters generated a wealth of grain boundaries and heterointerfaces, which, in turn, induced robust interfacial electron transfer properties, facilitated the immersion of electrolyte ions, and provided additional A‐C interfacial active sites for the HER. Results from HAADF‐STEM imaging combined with energy‐dispersive X‐ray spectroscopy (STEM‐EDX) revealed uniform distribution of Co, Pd, and S elements within the PdCo‐Co_3_S_4_ nanocages (Figure 1f), and the measured atomic distribution is 51.13%, 3.58%, and 45.29%, respectively (Figure S1, Supporting Information).

In the X‐ray diffraction (XRD) of the PdCo‐Co_3_S_4_ nanocages, all diffraction peaks were attributed to Co_3_S_4_ (JCPDS No.73‐1703), Pd (JCPDS No. 72–0710), and Co (JCPDS No. 70–2633), indicating the formation of heterogeneous PdCo‐Co_3_S_4_ composite material (Figure 2a). Furthermore, the increase in the concentration of 2‐methylimidazole could potentially alter the coordination environment of the metal centers and provide steric hindrance during the pyrolysis process, leading to potential aggregation, which might result in the growth of Co_3_S_4_ and Pd/Co along different directions and an increase in interlayer spacing, consistent with the analysis of different crystal planes observed in STEM. From the results of BET and BJH analyses, it can be observed that PdCo‐Co_3_S_4_ nanocages exhibit a commendable surface area and a relatively high pore volume, indicating their mesoporous nature (Figure 2b). As is well‐known, a higher surface area and mesoporous structure provide an abundance of metal active sites, facilitating electron transfer between the electrolyte and the catalyst, thereby promoting the absorption and dissociation of water molecules.^[^ ^19^ ^]^ In addition, Raman spectroscopy reveals several peaks at ≈193, 472, 515, and 678 cm^−1^, corresponding to the F_2g_, E_g_, F_2g_, and A_1g_ modes of Co_3_S_4_, respectively (Figure 2c; Figure S2, Supporting Information). Interestingly, a redshift is observed in the A_1g_ mode peak dominated by the symmetric stretching of Co─S bonds at tetrahedral sites in PdCo‐Co_3_S_4_. This indicates that the introduction of Pd induces a change in the chemical state between Co and S, selectively affecting Co^2+^ at tetrahedral sites. This also confirms that the electron transfer between Pd and Co has modulated the Co d‐band center, enhancing the intrinsic catalytic activity.

The compositions and chemical states of Co_3_S_4_ and PdCo‐Co_3_S_4_ were probed by X‐ray photoemission spectroscopy (XPS). In Figure 2d, the S 2p spectrum displays two peaks at 161.5 and 162.7 eV, confirming the presence of Co_3_S_4_. The peaks at 164.5 and 169.1 eV are associated with O═S═O and sulfate bonds, respectively. As shown in Figure 2f, the Pd 3d_5/2_ and 3d_3/2_ peaks of CoPd‐Co_3_S_4_ can be deconvoluted into Pd^0^ and Pd^2+^ components. It is worth noting that a small amount of Pd^2+^ species may arise due to surface oxidation. The Co 2p spectrum for Co_3_S_4_ and PdCo‐Co_3_S_4_ can be attributed to Co(III) 2p_3/2_ and 2p_1/2_ peaks, as well as Co(II) 2p_3/2_ and 2p_1/2_ peaks (Figure 2e). Additionally, two broad peaks located at 785.4 and 802.9 eV correspond to satellites. The emergence of Co^0^ species stems from the reduction of free Co ions, resulting from Pd‐Co ion exchange, through the action of the reducing agent L‐ascorbic acid to Co metal. Conversely, the Co^2+^ and Co^3+^ species correspond to characteristic peaks associated with Co_3_S_4_ material, indicating the presence of the primary phase Co_3_S_4_ and the formation of defective Co_3‐x_S_4_ species. The binding energies of Co 2p_3/2_, 2p_1/2_, and the two satellite vibrations align well with the characteristic peaks of reported Co_3_S_4_, indicating that the valence states and ion distribution in PdCo‐Co_3_S_4_ are similar to Co_3_S_4_. It is noteworthy that the Co 2p XPS peak of PdCo‐Co_3_S_4_ shifts to lower binding energy compared to Co_3_S_4_, indicating electron transfer from Pd to Co. This suggests that the surface atomic defects caused by heterojunction edge dislocations lead to the transfer of electrons from Pd to Co on the surface of PdCo‐Co_3_S_4_ nanocages. The electron transfer from Pd to Co results in a downward shift of the d‐band center of Co relative to the Fermi level (E_f_) and an upward shift of the d‐band center of Pd. According to the d‐band center theory, the downward shift of the d‐band center of Co enhances the adsorption of Co sites for H_2_O, while the upward shift of the d‐band center of Pd weakens the adsorption of Pd sites for H*, both of which are beneficial for enhancing the activity of alkaline HER.

**Figure 3:**
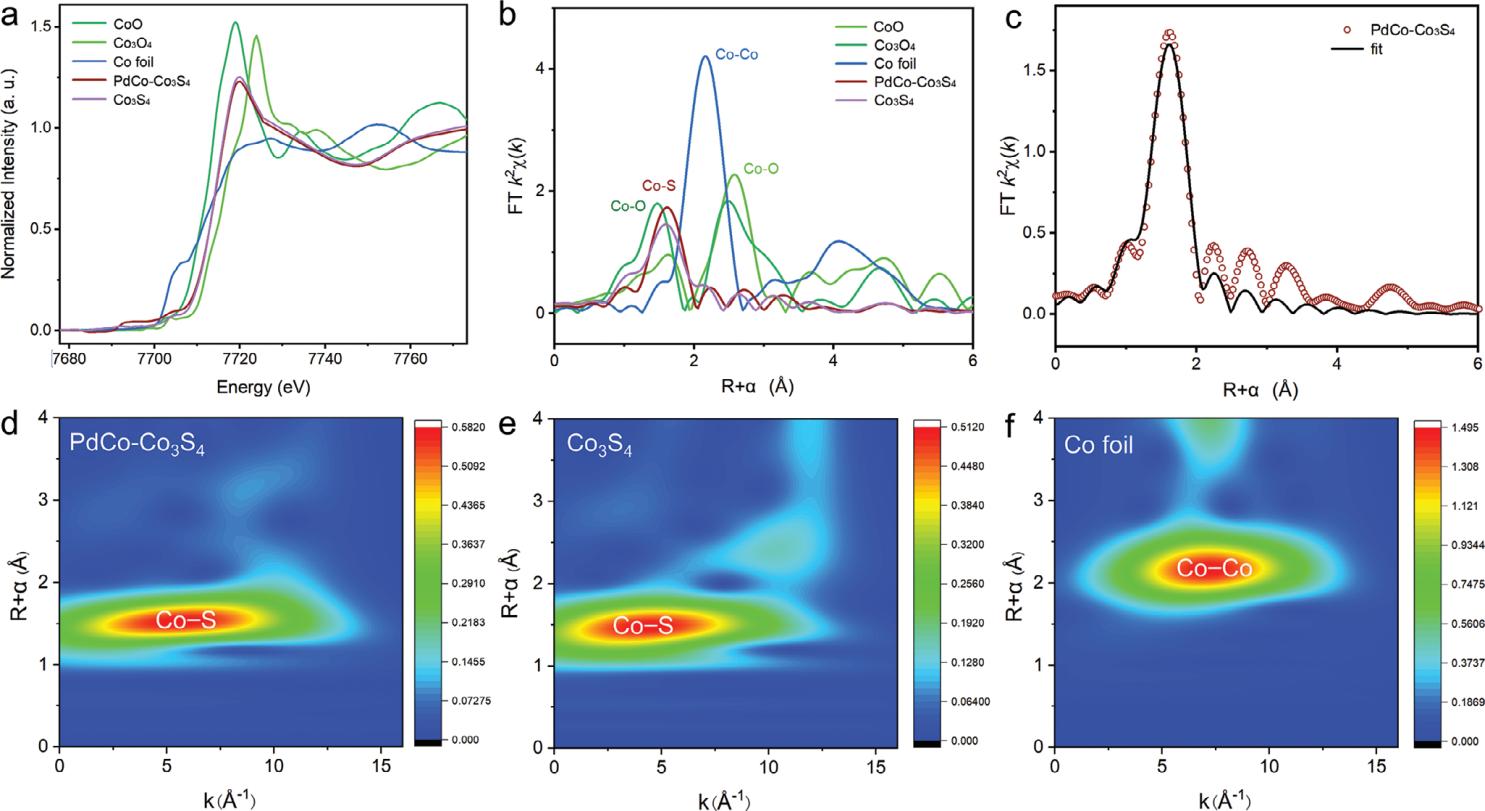
Chemical state and coordination structure analysis. a) Normalized XANES spectra and b) Fourier transformed EXAFS spectra at Co K‐edge of the Co foil, CoO, Co_3_O_4_, Co_3_S_4_, and PdCo‐Co_3_S_4_. c) Co K‐edge EXAFS fitting curve on PdCo‐Co_3_S_4_ in R‐space. WT‐EXAFS of d) PdCo‐Co_3_S_4_, e) Co_3_S_4_, and f) Co foil at the Co K‐edge.

Utilizing X‐ray absorption spectroscopy (XAS) provided essential insights into the electronic and local structural features of the samples. The Co K‐edge X‐ray absorption near‐edge structure (XANES), as depicted in **Figure** **3**a, showcased a higher energy shift for the Co K‐edge in PdCo‐Co_3_S_4_ compared to CoO, yet lower than that of Co_3_O_4_. This indicates that upon the formation of the PdCo‐Co_3_S_4_ heterostructure, the average valence state of Co ranged between +2 and +3. PdCo‐Co_3_S_4_ exhibited a Co─S coordination structure akin to Co_3_S_4_, albeit with a slightly reduced white‐line intensity, suggesting both the presence of Co_3_S_4_ and the interaction between Co_3_S_4_ and PdCo. In addition, the Fourier‐transformed extended X‐ray absorption fine structure (FT‐EXAFS) of PdCo‐Co_3_S_4_ in Figure 3b resembles Co_3_S_4_, yet significantly differs from Co foil, Co_3_O_4_, and CoO. Therefore, the peak at 1.62 Å can be attributed to the Co─S bond.^[^ ^20^ ^]^ Concurrently, employing Co foil as a reference, EXAFS fitting of PdCo‐Co_3_S_4_ within the 1.0−3.0 Å range revealed a coordination number of 3.7 for the Co─S bond, while in Co foil, the coordination number for the Co─Co bond is 12 (Figure 3c; Figure S3 and Table S1, Supporting Information). Furthermore, wavelet transforms were applied to the EXAFS spectra, as they afford a clear elucidation of the changes in coordination environments at radial distances and in K‐space resolution.^[^ ^21^ ^]^ Figures 3d–f present the wavelet transform (WT) profiles of the first shell with optimal resolution based on Morlet wavelets for Co foil, Co_3_S_4_, and PdCo‐Co_3_S_4_. In the WT contour plot of PdCo‐Co_3_S_4_, the peak intensity at 5.60 Å corresponds to the Co─S contribution, akin to that of Co_3_S_4_, yet notably distinct from other contributions (Figure S4, Supporting Information).

### 2.2 Electrocatalytic HER Performance and Insight into the Catalytic Activity

**Figure 4:**
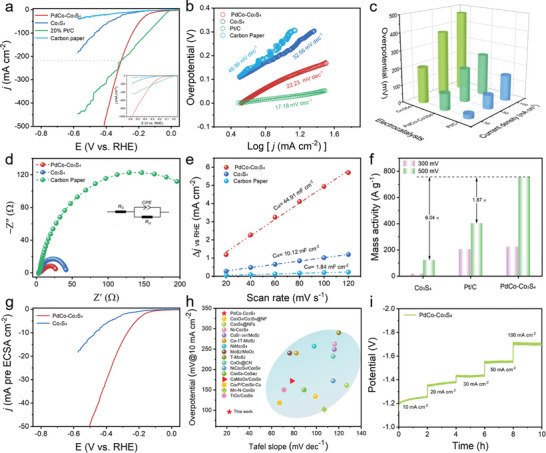
Evaluation of Electrocatalytic HER activity. a) Polarization (LSV) curves and b) corresponding Tafel plots of PdCo‐Co_3_S_4_, Co_3_S_4_, and Pt/C catalysts in 1 m KOH electrolyte. c) Overpotentials of various electrodes at the corresponding current densities; d) EIS Nyquist plots. e) C_dl_ values for PdCo‐Co_3_S_4_, and Co_3_S_4_. f) Mass activity for PdCo‐Co_3_S_4_, Pt/C, and Co_3_S_4_ at overpotentials of 300 and 500 mV. g) ECSA‐normalized LSV curves of PdCo‐Co_3_S_4_, and Co_3_S_4_. h) Comparison of the HER activities of various Co‐based non‐noble metal catalysts in 1.0 m KOH alkaline solutions. i) Multi‐step chronopotentiometry tests at variable current density.

In order to elucidate the excellent electrochemical HER properties arising from the phase engineering, we conducted electrocatalytic performance tests on the prepared materials in a 1.0 m KOH electrolyte. **Figure** **4**a illustrates the polarization (LSV) curves of the samples, where PdCo‐Co_3_S_4_ exhibits a remarkable HER performance, with its current density rapidly increasing with increasing potential. PdCo‐Co_3_S_4_ achieves an overpotential of only 96 mV to reach 10 mA cm^−2^ (Figure 4c), significantly lower than that of Co_3_S_4_ (206 mV) and carbon paper (275 mV). Importantly, PdCo‐Co_3_S_4_ requires only 306 mV to achieve 217 mA cm^−2^, surpassing 20% Pt/C, thus demonstrating exceptional electrocatalytic performance. Tafel curves for all electrodes were produced by fitting the LSV curves to further examine the HER kinetics for each electrode (Figure 4b). Among these, PdCo‐Co_3_S_4_ exhibits the smallest Tafel value (22.23 mV dec^−1^), indicating superior kinetics and faster electron transfer capability compared to Co_3_S_4_ (32.56 mV dec^−1^) and carbon paper (48.99 mV dec^−1^). Furthermore, the Tafel slopes of PdCo‐Co_3_S_4_ are close to 30 mV dec^−1^, indicating that the kinetics of the HER process are primarily controlled by the hydrogen evolution step (M + H_2_O + e^−^ → MH_ads_+OH^−^, where M represents the catalyst, i.e., the Volmer‐Tafel mechanism).^[^ ^22^ ^]^ Furthermore, the multiphase cluster‐crystalline‐amorphous defect interface provides additional electrochemical active regions and catalytic active sites, thereby enhancing the kinetics of HER.^[^ ^23^ ^]^

The charge transfer resistance (R_ct_) of PdCo‐Co_3_S_4_ determined by electrochemical impedance spectroscopy (EIS) (Figure 4d) is significantly lower than that of Co_3_S_4_ and bare carbon paper, indicating that the former accelerates charge transfer and exhibits outstanding reaction kinetics. This is advantageous for the binding of electrons and adsorbed hydrogen (H_ads_), further enhancing the performance of the HER.^[^ ^24^ ^]^ Therefore, refining the interface with fully exposed clusters to achieve perfect electronic interactions contributes to improving the intrinsic conductivity and catalytic performance of the material.

To further investigate the intrinsic performance of the prepared PdCo‐Co_3_S_4_ nanocages, the electrochemical surface area (ECSA) was calculated according to the double‐layer capacitance (C_dl_). The value of C_dl_ is further fitted by measuring CV curves (Figure S5, Supporting Information) at different scan rates. The measurement of the ECSA, which is directly proportional to the double‐layer capacitance (C_dl_), was undertaken to ascertain the intrinsic performance of the catalyst (Figure 4e). Notably, PdCo‐Co_3_S_4_ exhibited the highest ECSA value at 44.91 mF cm^−2^. This substantial increase in ECSA, attributed to its significantly larger specific surface area, surpassing that of Co_3_S_4_ by more than fourfold, can be attributed to the open and hollow nanocages structure of PdCo‐Co_3_S_4_. This unique structural feature contributes to an extended electrocatalyst‐electrolyte interface, thereby enhancing the exposure of active sites and promoting heightened electrocatalytic activity.^[^ ^25^ ^]^

Furthermore, the mass activity of PdCo‐Co_3_S_4_ exhibited remarkable enhancements, surpassing that of Co_3_S_4_ by factors of 10.4 and 6.04 at overpotentials of 300 and 500 mV (Figures 4f). This underscores the beneficial influence of the nanocages architecture in augmenting the intrinsic activity of each accessible active site. Impressively, when the specific activity of the samples was normalized by their ECSA to eliminate the influence of active site quantity, PdCo‐Co_3_S_4_ exhibited substantially superior normalized HER activity compared to other controls (Figure 4g), which demonstrates that the incorporation of Pd‐Co heterogeneous clusters and the formation of defects resulting in surface restructuring can profoundly enhance intrinsic activity.

Notably, the outstanding HER performance promoted by Pd‐Co heterogeneous clusters into Co_3_S_4_ can compete with or even outperform most of the previously reported HER catalysts in 1.0 m KOH alkaline solutions (Figure 4h; Table S2, Supporting Information). Meanwhile, the stability of PdCo‐Co_3_S_4_ can be maintained by multi‐step chronopotentiometry tests at variable current density (Figure 4i). The constructed electrolyzer demonstrated outstanding stability in long‐term operation, even at 100 mA cm^−2^, the catalytic performance declined only slightly after 20 h of electrolysis (Figures S6, Supporting Information). The SEM, and XRD analyses following stability testing revealed the well‐preserved nanocage morphology and structure of PdCo‐Co_3_S_4_, demonstrating excellent stability (Figures S7 and S8, Supporting Information). Furthermore, the nanocluster island effect and heterogeneous A‐C interface contribute to stabilizing the surface‐active species, ensuring efficient HER. The intact A‐C heterostructure observed via STEM further underscores the exceptional structural stability of PdCo‐Co_3_S_4_ (Figure S9, Supporting Information). These findings confirm the outstanding intrinsic catalytic activity and corrosion resistance of the PdCo‐Co_3_S_4_ electrode for HER in alkaline solutions.

### 2.3 Solar‐Driven Alkaline Seawater Hydrogen Evolution Systems

**Figure 5:**
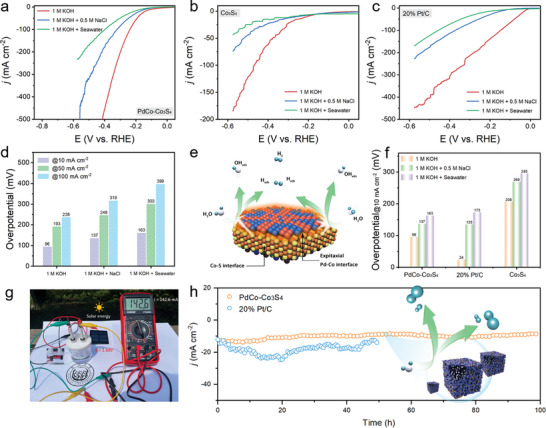
Natural/Simulated alkaline seawater hydrogen evolution. Polarization Curves of a) PdCo‐Co_3_S_4_, b) Co_3_S_4_, and c) 20% Pt/C in varied electrolytes during the HER. d) Overpotential requirements for PdCo‐Co_3_S_4_ at 10, 50, and 100 mA cm^−2^ in different electrolytes. e) Schematic of the HER steps in basic medium. f) Overpotential requirements for various catalysts at 10 mA cm^−2^ in different electrolytes. g) Solar‐driven water electrolyzer. h) Extended Durability Assessment of a Self‐Sustaining Seawater Electrolysis System.

Inspired by the outstanding electrocatalytic performance of the PdCo‐Co_3_S_4_ electrode in alkaline water, further investigations were conducted to assess its electrocatalytic HER performance in simulated alkaline seawater and natural alkaline seawater electrolytes. Natural seawater (pH 7.8) was collected from the East China Sea (Figure S10, Supporting Information). The HER catalytic performance of the catalyst in both natural seawater and simulated alkaline seawater is depicted in **Figure** **5**a–c. As anticipated, PdCo‐Co_3_S_4_ exhibited excellent catalytic performance in the complex alkaline seawater electrolyte (Figure 5d). It achieved a low overpotential of 163 mV for hydrogen evolution at 10 mA cm^−2^, which is much lower than that of Co_3_S_4_ (295 mV), and 20% Pt/C (173 mV) (Figure 5f). This indicates the practical potential of PdCo‐Co_3_S_4_ for electrolysis based on natural seawater hydrogen production. Furthermore, in the 1.0 m KOH + seawater electrolyte, the electrocatalyst displays some activity degradation due to the burial of active sites and electrode poisoning by pollutants or small insoluble precipitates in natural seawater.

It is noted that the HER performance of the PdCo‐Co_3_S_4_ electrode in natural seawater was inferior to that in simulated alkaline seawater, which may be attributed to the presence of a substantial amount of unremoved Ca^2+^ and Mg^2+^ ions in natural seawater.^[^ ^5b^ ^]^ These ions tend to generate precipitates on the electrode surface during the HER process, thereby covering active sites and diminishing the catalyst's performance.^[^ ^4^ ^]^ Impressively, this configuration remained highly stable (Figure S11, Supporting Information), with no significant activity degradation, even after 100 h of operation at 10 mA cm^−2^. Consequently, it exhibits exceptional corrosion resistance in alkaline seawater electrolysis, significantly surpassing commercial Pt/C catalysts (Figure 5h). The slight positive shift can be attributed to surface blockage caused by the formation of irreversible intermediates and bubble attachment, leading to a reduction in the effective contact area between the electrode and the electrolyte.^[^ ^26^ ^]^ In conclusion, the induction of surface amorphization in electrocatalysts represents a promising strategy for enhancing the exposure of catalytically active sites, elevating intrinsic catalytic activity, and improving electrochemical stability.

Given its exceptional electrocatalytic performance, we constructed a solar‐driven seawater electrolysis system (Figure 5g) powered by solar cells to showcase the superior practical utility of PdCo‐Co_3_S_4_ nanocages under real solar irradiation conditions. This can be attributed to the nanoscale island‐like structure of PdCo clusters and the A‐C heterostructure of Co_3_S_4_ nanocages, which embodies the “moving but not aggregating” design of metal active sites (Figure 5e). These findings indicate that PdCo‐Co_3_S_4_ nanocages demonstrate outstanding seawater splitting performance, rendering them a highly promising candidate for use in solar energy storage and the production of solar to hydrogen.

### 2.4 Density Functional Theory (DFT) Calculations

**Figure 6:**
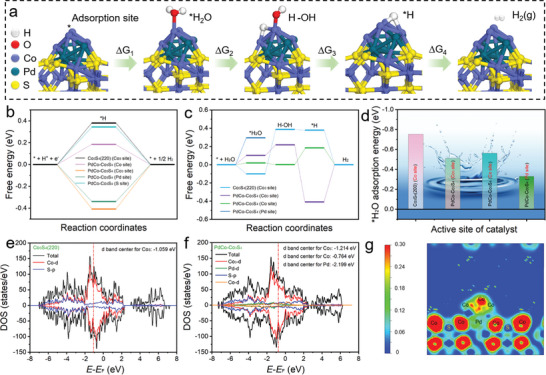
Theoretical investigations. a) Process of hydrogen production on PdCo‐Co_3_S_4_. b) Calculated free energy diagram of H adsorption for PdCo‐Co_3_S_4_. c) Calculated free energy of HER intermediates at zero potential. d) Calculated H_2_O adsorption energy. e,f) Electronic density of states (DOS), and d band center. g) Electron localization function (ELF) maps.

The first‐principles DFT calculations were carried out to elucidate the origin of the remarkable HER activity of PdCo‐Co_3_S_4_ heterostructure on the theoretical level. From the obtained Tafel slope, it can be inferred that the adsorption/desorption of water and the adsorption mechanism of hydrogen on the catalyst follow the Volmer‐Tafel pathway. Consequently, the free energy was computed according to this reaction pathway (**Figure** **6**a). The adsorption of reaction intermediates on the surfaces of PdCo‐Co_3_S_4_ and Co_3_S_4_ is illustrated in Figures S12 and S13 (Supporting Information). The intrinsic catalytic activity of the catalyst for the HER is typically assessed in terms of the hydrogen adsorption‐free energy (∆G_H*_).^[^ ^27^ ^]^ An ideal catalyst should exhibit an optimal ∆G_H*_ value close to 0 eV, indicating a suitable strength of H adsorption/desorption during the catalytic process.^[^ ^28^ ^]^ In this study, PdCo‐Co_3_S_4_ and Co_3_S_4_ (220) were employed as models (Figure S14, Supporting Information) to investigate potential adsorption sites for the H* intermediate with various active atoms. The ΔG_H*_ value for Co sites on the clusters was found to be 0.18 eV, which is closer to 0 eV compared to other sites (Figure 6b). Furthermore, the ΔG_H*_ value for Pd sites on PdCo‐Co_3_S_4_ was −0.33 eV, closer to 0 eV than the ΔG_H*_ value for Co sites on Co_3_S_4_ (220) (0.38 eV). This could be attributed to the influence of Pd introduction on the electronic structure of the Co sites. It indicates that the reactive O─H bond cleavage could significantly increase the H* concentration at the PdCo‐Co_3_S_4_ interface, thereby accelerating the alkaline HER process.

The calculation of water molecule adsorption energy (ΔE_H2O_) on the catalyst surface is highly necessary as it directly impacts the rate of catalytic reactions.^[^ ^29^ ^]^ Generally, the alkaline HER has a two‐step process: the Volmer step including the H_2_O adsorption and H_2_O dissociation along with the cleavage of O─H bonds to form H atoms, and the Heyrovsky step or the Tafel step corresponding to the H_2_ generation.^[^ ^30^ ^]^ Thus, we further verify the origin of the activity on PdCo‐Co_3_S_4_ by evaluating the free energies of the H_2_O adsorption, H_2_O dissociation (ΔG_OH−H_), and H adsorption (ΔG_H_) on the surfaces of the Co_3_S_4_ (Co_3_ sites), PdCo‐Co_3_S_4_ (Co_3_ sites), PdCo‐Co_3_S_4_ (Pd sites), and PdCo‐Co_3_S_4_ (Co sites). In Figure 6d, the Co_3_S_4_ (Co_3_ sites) has the strongest ΔE_H2O_ value (−0.75 eV), implying that the abundant surface area and mesoporous structure are favorable to the strong adsorption capacity for H_2_O on Co_3_S_4_ nanocage surface. The PdCo‐Co_3_S_4_ (Co sites), containing the distinctive hollow nano‐cubic structure of Co_3_S_4_ as a promoter for H_2_O adsorption, shows integrated adsorption energy (−0.56 eV), which is superior to the PdCo‐Co_3_S_4_ (Pd sites) (−0.33 eV) and PdCo‐Co_3_S_4_ (Co_3_ sites) (−0.51 eV).

The key reaction steps of the alkaline HER and the optimal configuration of relevant species on Co_3_S_4_ (220) and PdCo‐Co_3_S_4_ are shown in Figure 6c as well. As discussed in previous reports,^[^ ^31^ ^]^ the sluggish kinetics of the HER in alkaline solution can be attributed to the initial adsorption and dissociation of water molecules on the catalyst surface. As shown in Figure 6c, the Gibbs free energy change associated with the formation of H─OH intermediates in the water dissociation step (ΔG_H─OH_) was found to be energetically favorable for both Co_3_ sites and Co sites, suggesting a rate‐limiting role, and hence it was applied as an activity descriptor. The ΔG_H─OH_ value of Co_3_S_4_ (Co_3_ sites) was as high as 0.38 eV, and reduced to −0.02 eV for PdCo‐Co_3_S_4_ (Co sites), suggesting a promoted water dissociation process. From a thermodynamic point of view, PdCo‐Co_3_S_4_ (Co sites) should exhibit greater alkaline HER activity, in agreement with our electrochemical experiments. The formation of cluster‐decorated amorphous/crystalline heterogeneous interfaces increases the number of hydroxyl adsorption active sites on the PdCo‐Co_3_S_4_, thereby promoting the cleavage of the HO─H bond and the formation of H intermediates. Therefore, the activation barrier for hydrolysis is notably reduced at the heterogeneous interface, accelerating the HER kinetics.

Furthermore, the changes in the electronic structure of the PdCo‐Co_3_S_4_ amorphous/crystalline heterogeneous interface were studied using the Density of States (DOS). The d‐band center of the Co_3_ site in the initial Co_3_S_4_ (220) (−1.059 eV) is relatively close to the Fermi level, while the d‐band center of the Co_3_ site in PdCo‐Co_3_S_4_ formed after Pd doping (−1.214 eV) shifts downward, resulting in weaker activity, whereas the d‐band center of the Co site in the loaded PdCo clusters (−0.764 eV) is closer to the Fermi level, making it more active. Therefore, the Co in the loaded PdCo clusters becomes the new active site (Figure 6e,f). These changes in the d‐band centers resulted in reduced adsorption strength for hydrogen intermediates, thus expediting the desorption process during the HER.^[^ ^32^ ^]^ In summary, the incorporation of PdCo clusters serves not only as high‐activity sites but also as modulators of Co_3_S_4_ electronic structure, enhancing electrical conductivity and charge transfer kinetics, lowering reaction barriers, and promoting HER. Furthermore, the analysis of the Electron Localization Function (ELF) provides additional support for the stability of PdCo‐Co_3_S_4_, offering insights into the material bonding characteristics.^[^ ^33^ ^]^ The ELF value between Co and Pd atoms is ≈0.05, indicating a metallic bond characteristic in Co─Pd interactions (Figure 6g). The robust interaction between the PdCo nanoclusters and the Co_3_S_4_ surface significantly impedes the migration and aggregation of active species, resulting in improved dispersion and uniformity of PdCo nanoclusters throughout the synthesis process.

## 3 Conclusion

In summary, we have employed an ion‐exchange strategy to fabricate a novel PdCo cluster‐Co_3_S_4_ heterogeneous nanocage with distinct amorphous‐crystalline phase boundaries for efficient and stable electrocatalytic seawater splitting to produce hydrogen. The optimized PdCo‐Co_3_S_4_ exhibits an overpotential of 96 mV at a current density of 10 mA cm^−2^ and a Tafel slope of 22.23 mV dec^−1^ in 1.0 m KOH, demonstrating excellent catalytic activity and long‐term stability in naturally occurring alkaline seawater solutions. These results are achieved through the rich electrocatalytic active centers provided by the A‐C heterogeneous interface, enhanced surface permeability, high electron conductivity of PdCo clusters, and the porous 3D structure of the nanocage. The amorphous portion of the material offers a wealth of active sites, including defects and unsaturated coordination sites, while the crystalline segment, distinguished by its high electron conductivity, ensures rapid charge transfer. The distinctive heterointerface formed within the A‐C heterostructure effectively mitigates the weaknesses associated with a singular amorphous or crystalline heterostructure, markedly shortening both ion and electron diffusion pathways, thereby enhancing the kinetics of the HER. Theoretical calculations indicate that the constructed A‐C heterostructures can significantly reduce the Gibbs free energy, providing a diversified range of active sites, and thereby enhancing HER activity. This study offers a potential solution to the challenges in electrochemical water splitting for HER, contributing to the United Nations Sustainable Development Goal 7: Affordable and Clean Energy.

## 4 Experimental Section

### Synthesis of the PdCo‐Co_3_S_4_ Heterostructure Nanocages

To synthesize the hollow nanocages in PdCo‐Co_3_S_4_, 0.15 g of Co_3_S_4_ nanoboxes was initially dispersed in 50 mL of EtOH, and ultrasonicated for 5 min to yield a homogeneous slurry. Subsequently, 0.1 mm of sodium tetrachloropalladate (Na_2_PdCl_4_) was introduced into the solution, followed by stirring at 60 °C for 180 min. The reaction mixture was then allowed to cool to room temperature (25 °C), and the resulting precipitate was harvested after several cycles of ethanol washing and centrifugation. Finally, L‐ascorbic acid solution (0.2 m) was added and stirred for 30 min. The collected material was subsequently dried at 60 °C.

### Electrochemical Measurements

All electrochemical measurements were conducted within a three‐electrode cell using an electrochemical workstation CHI 760E. The working electrode consisted of carbon paper (CP) cut to 0.5 × 0.5 cm^2^ loaded with catalyst, in which electrocatalyst powder ink was prepared using a mixture of 0.70 mL deionized water, 0.25 mL ethanol, 0.05 mL Nafion solution, and 10 mg catalyst, and then sonicated for 30 min. The ink was then uniformly applied on a CP with a catalyst loading of 1 mg cm^−2^, the CP was used as the working electrode, the Pt net as the counter electrode, Hg/HgO as the reference electrode, and 1.0 m saturated KOH aqueous solution was used. All measured potentials were referred to the reversible hydrogen electrode (RHE) using the following equation: *E*(RHE) = *E*(Hg/HgO) + 0.059 × pH + 0.098 V, and the current densities (*j*) were normalized by geometric surface area. The frequency setting range of the EIS test is from 100 kHz to 0.01 Hz. C_dl_ was estimated from the CV method at various scan rates (20, 40, 60, 80, 100, 120 mV s^−1^) in the non‐Faraday zone, and C_dl_ was given in the following equation: Δ*j* = *j*_anodic_ – *j*_cathodic_ = 2 × v × C_dl_. All the potential was recorded without *iR*‐correction.

## Conflict of Interest

The authors declare no conflict of interest.

## Author Contributions

The manuscript was written through the contributions of all authors.

## Supporting information

Supporting Information

## Data Availability

The data that support the findings of this study are available from the corresponding author upon reasonable request.
