# Supplementary material for: Constructing Amorphous‐Crystalline Interfacial Bifunctional Site Island‐Sea Synergy by Morphology Engineering Boosts Alkaline Seawater Hydrogen Evolution
Source: Adv Sci (Weinh). 2024 Mar 18;11(24):2309927. doi: 10.1002/advs.202309927 (PMC11199995; doi:10.1002/advs.202309927)
Supplement: Supplementary file 1 — Supporting Information [file ADVS-11-2309927-s001.pdf]

ADVANCED SCIENCE
Open Access

Supporting Information

for *Adv. Sci.*, DOI 10.1002/advs.202309927

Constructing Amorphous-Crystalline Interfacial Bifunctional Site Island-Sea Synergy by Morphology Engineering Boosts Alkaline Seawater Hydrogen Evolution

Pengliang Sun, Xiong Zheng, Anran Chen, Guanghong Zheng, Yang Wu, Min Long, Qingran Zhang and Yinguang Chen*

Supporting Information

Constructing Amorphous-Crystalline Interfacial Bifunctional Site Island-Sea Synergy by Morphology Engineering Boosts Alkaline Seawater Hydrogen Evolution

Pengliang Sun [a], *Xiong Zheng* [a,b,]*, *Anran Chen* [c], *Guanghong Zheng* [a], *Yang Wu* [a], *Min Long* [a], *Qingran Zhang* [a,b], *and Yinguang Chen* [a,b]

[a] State Key Laboratory of Pollution Control and Resource Reuse, School of Environmental Science and Engineering, Tongji University, Shanghai 200092, P.R. China.

[b] Shanghai Institute of Pollution Control and Ecological Security, Shanghai 200092, P.R. China.

[c] School of Materials and Energy, Yunnan University, Kunming 650091, P.R. China.

* Corresponding authors.

Tel.: +86 21 65981263

Fax: +86 21 65986313

E-mail addresses: xiongzheng@tongji.edu.cn (X. Zheng).

Experimental Section

Chemicals

Cobalt nitrate hexahydrate ($Co(NO_3)_2{\cdot}6H_2O$, 99.99%), 2-Methylimidazole ($C_4H_6N_2$, ≥99%), sodium tetrachloropalladate (Na_2PdCl_4, 99.9%), and thioacetamide (CH_3CSNH_2, AR) were purchased from Shanghai Aladdin Co., Ltd. Ethanol (EtOH, 99%), sodium chloride (NaCl, 99%), and NaOH (GR) were provided by Shanghai Macklin Biochemical Technology Co., Ltd. Cetyltrimethylammonium bromide (CTAB, 99%) were ordered from Sinopharm Chemical Reagent Co., Ltd. (Shanghai, China). All the chemicals were used without further purification.

Preparation of ZIF-67 Nanocubes Precursors

The preparation of ZIF-67 templating precursor follows the established protocol as detailed below: Initially, cobalt nitrate hexahydrate (1.5 mM) and cetyltrimethylammonium bromide were dissolved in deionized water (10 mL), yielding a homogeneous solution. Subsequently, 2-methylimidazole (5.4 g) was dissolved in pure water (40 mL) to generate a transparent solution. The two solutions were then combined and vigorously stirred for 15 minutes, resulting in the formation of a deep blue solution. The ensuing precipitate was collected via centrifugation, subjected to multiple ethanol washes, and dried overnight at 60 °C.

Preparation of Hollow nanoboxes in Co_3S_4

To prepare the hollow nanoboxes within Co_3S_4, 25 mg of ZIF-67 particles were first redispersed in 20 mL of ethanol and subjected to 30 minutes of ultrasonication, resulting in a homogeneous solution. Subsequently, 2 mL of thioacetamide (0.1875 mM in ethanol) was added to the solution with continuous stirring and maintained at 90 °C for 1 hour. After naturally

cooling to room temperature, the black precipitate was collected via repeated washing and centrifugation steps. It was thoroughly rinsed with deionized water and ethanol, followed by drying at 60 °C.

Electrochemical Measurements

All electrochemical measurements were conducted within a three-electrode cell using an electrochemical workstation CHI 760E. The working electrode consisted of carbon paper (CP) cut to 0.5 × 0.5 cm^2 loaded with catalyst, in which electrocatalyst powder ink was prepared using a mixture of 0.70 mL deionized water, 0.25 mL ethanol, 0.05 mL Nafion solution, and 10 mg catalyst, and then sonicated for 30 min. The ink was then uniformly applied on a CP with a catalyst loading of 1 mg cm^{-2}, the CP was used as the working electrode, the Pt net as the counter electrode, Hg/HgO as the reference electrode, and 1.0 M saturated KOH aqueous solution was used. All measured potentials were referred to the reversible hydrogen electrode (RHE) using the following equation: E(RHE) = E(Hg/HgO) + 0.059 × pH + 0.098 V, and the current densities (j) were normalized by geometric surface area. The frequency setting range of the EIS test is from 100 kHz to 0.01 Hz. C_{dl} was estimated from the CV method at various scan rates (20, 40, 60, 80, 100, 120 mV s^{-1}) in the non-Faraday zone, and C_{dl} was given in the following equation: $\Delta j = j_{anodic} - j_{cathodic} = 2 \times v \times C_{dl}$. All the potential was recorded without iR-correction.

Instruments and Characterizations

Morphology images were obtained by filed-emission scanning electron microscopy (JEOL JSM6701 FESEM) and the transmission electron microscopy (JEOL JEM-2800 TEM) with a scanning transmission electron microscopy (STEM) detector and energy-dispersive X-ray (EDX)

analysis. X-ray photoelectron spectroscopy (XPS) measurement was conducted on the ESCALAB 250XI photoelectron spectrometer (Thermo Fisher Scientific, USA) fixed with Al Kα 1846.6 eV anode. Raman spectra of the samples were recorded on a WITec system with a 532 nm excitation wavelength. X-ray powder diffraction (XRD, GBC MMA diffractometer, Cu Kα radiation, λ=0.15406 nm) was used to characterize the crystal structure. Nitrogen adsorption-desorption isotherms were recorded at −196 °C on a static volumetric instrument (Autosorb-6b, Quanta Chrome), after the sample vacuum degassed at 250 °C for 12 h. The specific surface area was calculated by the Brunauer– Emmett–Teller (BET) method. XAS tests were performed at the photoemission end-station at beamline BL10B of National Synchrotron Radiation Laboratory (NSRL) in Hefei, China. XAS data extracted and analyzed using the Athena and Artemis codes.

Electrochemical active surface area (ECSA)

The active surface area of each catalyst was estimated from their electrochemical capacitances, which can be measured using the simple cyclic voltammetry method. The current was measured in a narrow potential window where no faradaic process was observed. We sweep the potential between 0.54 and 0.64 V vs. RHE (HER) at each of six different scan rates (20, 40, 60, 80, 100, and 120 mV s^{-1}). By plotting the difference in current density (J) between anodic and cathodic sweeps (ΔJ) at a fixed potential against the scan rate, a linear trend is observed. The fitting slope is twice the double-layer capacitance (C_{dl}), which is linearly promotional to the ECSA. The specific capacitance for a flat surface is normally between 0.02-0.06 mF cm^{-2} (C_s=0.04 mF cm^{-2}). The calculation formula is as follows:

$$ECSA = \frac{C_{dl}(catalyst)\ mF\ cm^{-2}}{C_s \cdot per\ ECSA\ cm^{-2}}$$

These values of C_{dl} permit comparison of the relative surface activity of different electrodes, particularly in the same electrolyte.

Computational Details

We carried out all the DFT calculations in the Vienna *ab initio* simulation (VASP 5.4.4) code.[21] The exchange-correlation is simulated with PBE functional and the ion-electron interactions were described by the PAW method.[22,23] The vdWs interaction was included by using empirical DFT-D3 method.[24] The Co_3S_4 (220) surface and the Co_3S_4 (220) surface supported Pd_5Co_4 nanocluster were employed to simulate the adsorption and reduction of H_2O and HER. All nanocluster atoms and the atoms in upper two layers of these surface are allowed to move freely while the bottom two layers of Co_3S_4 (220) surface are fixed during the geometry optimization. The Monkhorst-Pack-grid-mesh-based Brillouin zone k-points are set as 1×2×1 for all periodic structure with the cutoff energy of 450 eV. The convergence criteria are set as 0.01 eV A^{-1} and 10^{-5} eV in force and energy, respectively.

The free energy calculation of species adsorption (ΔG) is based on following model.

$$\Delta G = \Delta E + \Delta E_{\mathrm{ZPE}} + \Delta H_{0\rightarrow T} - T\Delta S \qquad (1)$$

Herein ΔE, ΔE_{ZPE}, and ΔS respectively represent the changes of electronic energy, zero-point energy, and entropy that caused by adsorption of intermediate. The $\Delta H_{0\rightarrow T}$ refers to the change in enthalpy when heating from 0K to T K. The ΔE is calculated with the formula:

$$\Delta E = E_{\mathrm{sotal}} - (E_{\mathrm{adsobate}} + E_{\mathrm{substrate}}) \qquad (2)$$

Where the E_{total}, $E_{\mathrm{adsorbate}}$ and $E_{\mathrm{substrate}}$ are the DFT energy of the total system, the adsorbate and the substrate, respectively.

Supporting Figures

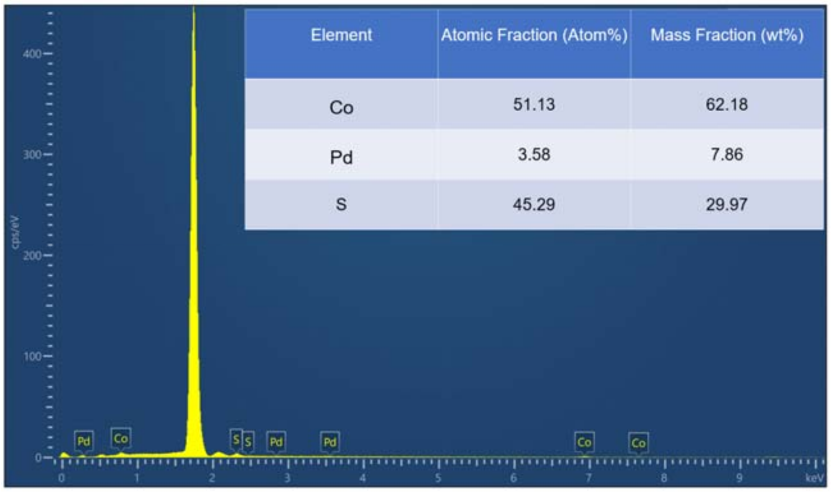

Figures S1. EDS spectra of PdCo-Co_3S_4 and the corresponding atomic and weight ratio of the element of Co, Pd, and S.

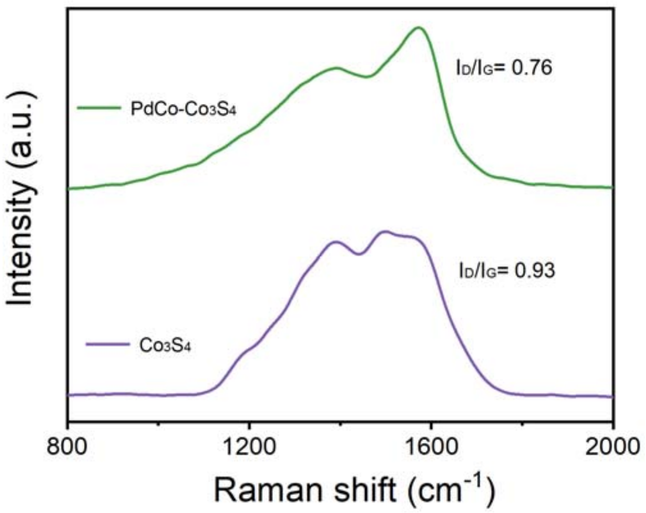

Figures S2. Raman spectra of the Co_3S_4 and PdCo-Co_3S_4.

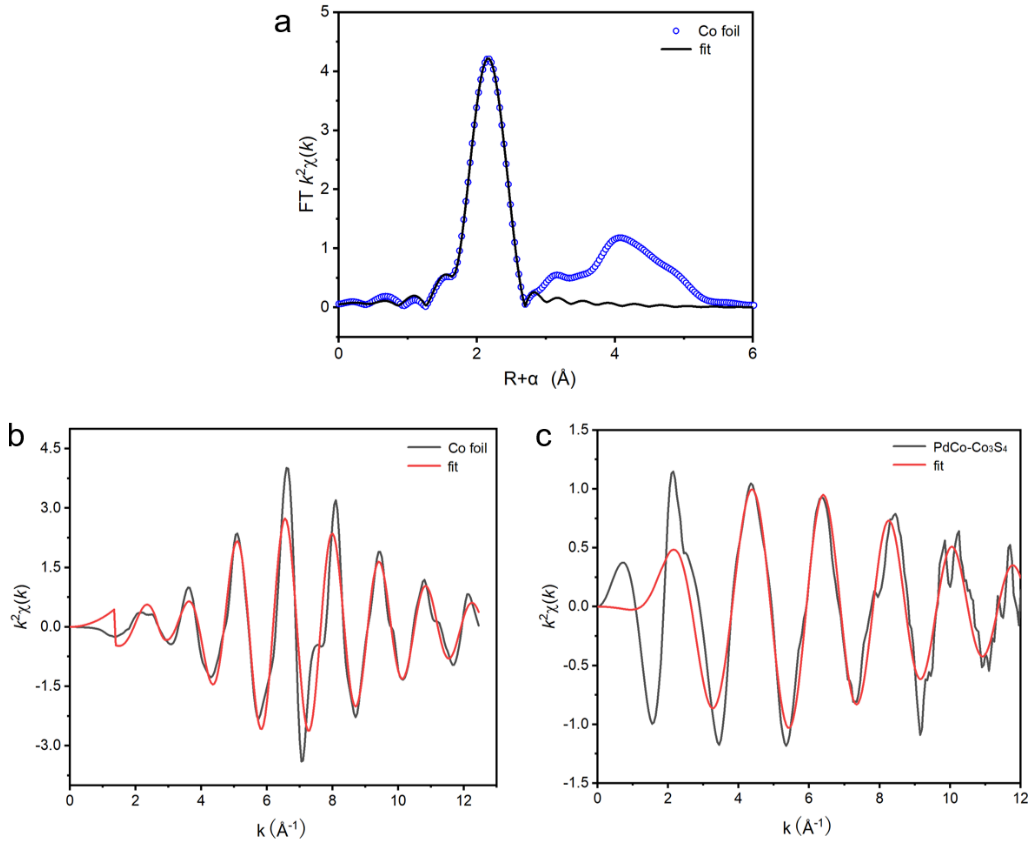

Figures S3. (a) The FT EXAFS fitting spectra of Co foil in R-space. Co L_3-edge EXAFS oscillation of (b) Co foil and (c) PdCo-Co_3S_4.

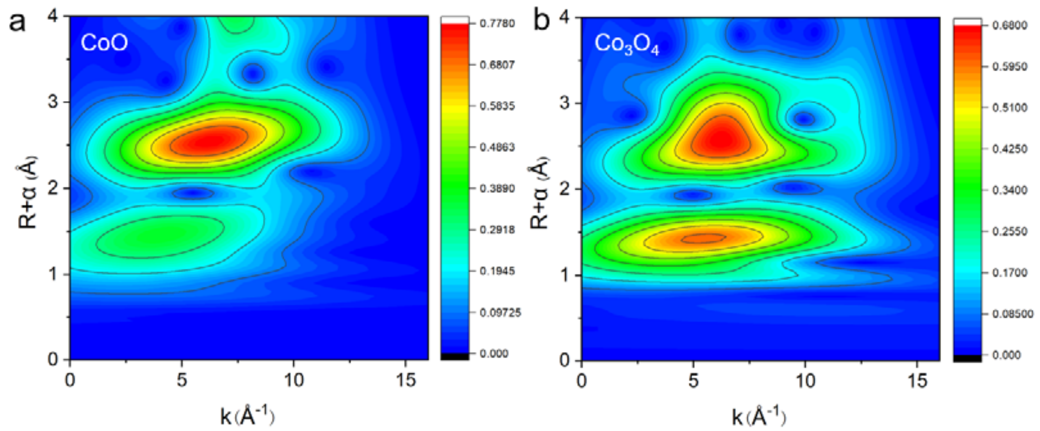

Figures S4. Wavelet transform (WT) of (a) CoO and (b) Co_3O_4.

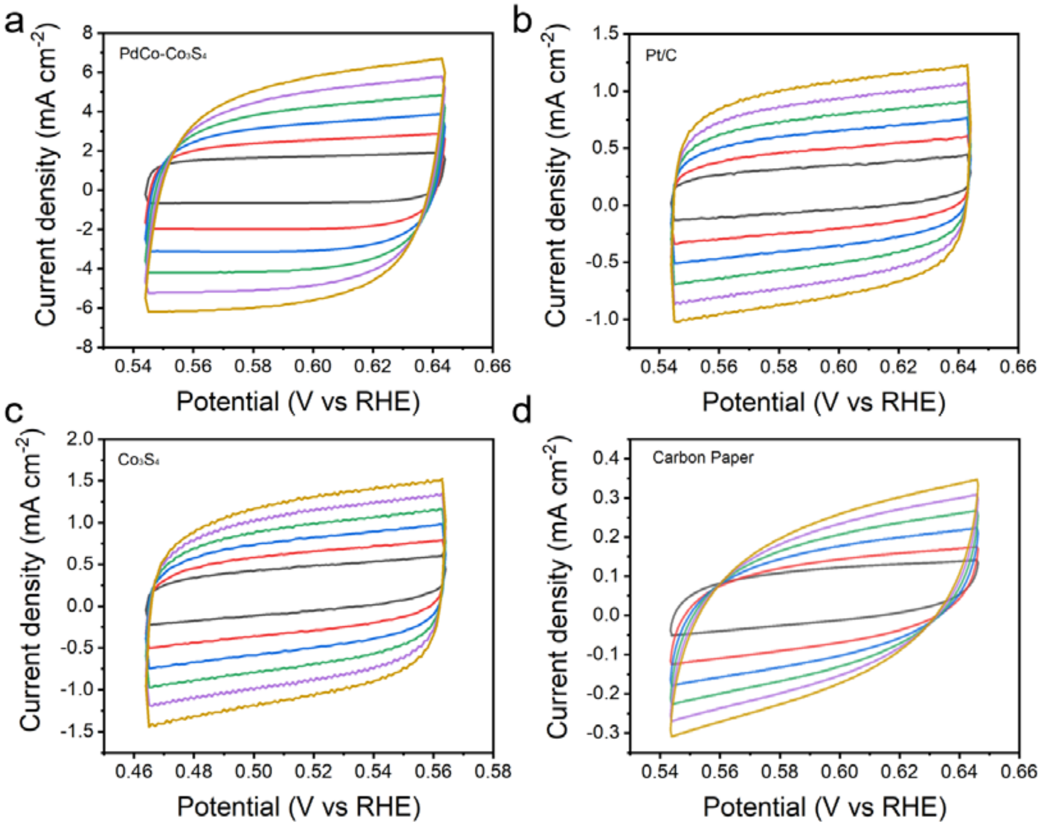

Figures S5. In the non-faradic capacitance current range of 20 to 120 mV s^{-1}, cyclic voltammograms of (a) PdCo-Co_3S_4, (b) Pt/C, (c) Co_3S_4, and (d) bare Carbon Paper were recorded.

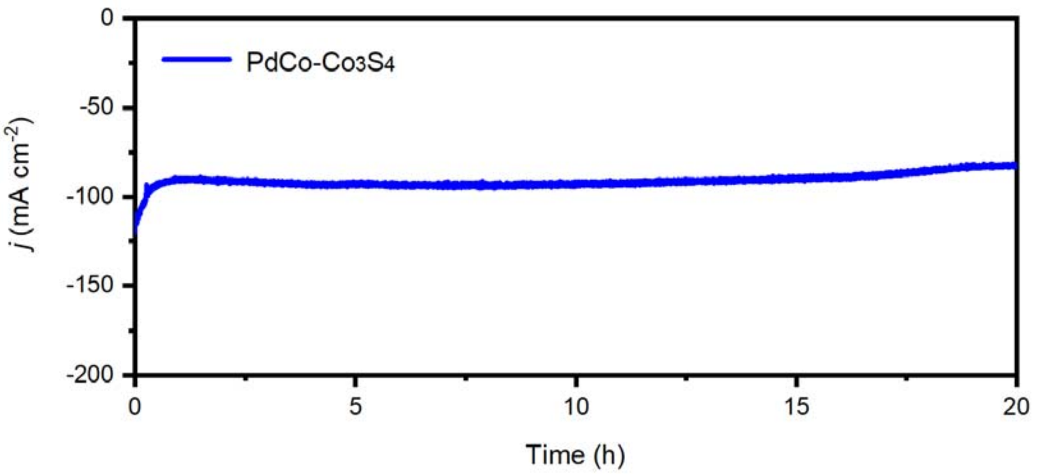

Figures S6. Chronoamperometric curve of PdCo-Co_3S_4 electrode.

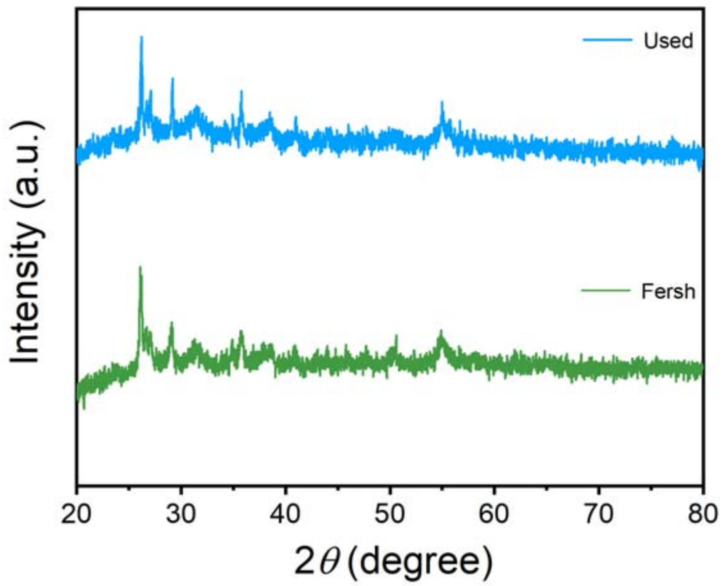

Figures S7. XRD pattern of the PdCo-Co_3S_4 before and after stability test.

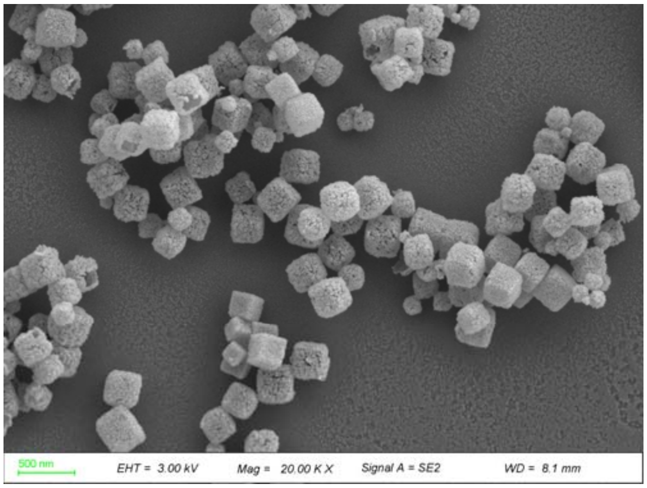

Figures S8. SEM images of PdCo-Co_3S_4 after the HER durability test.

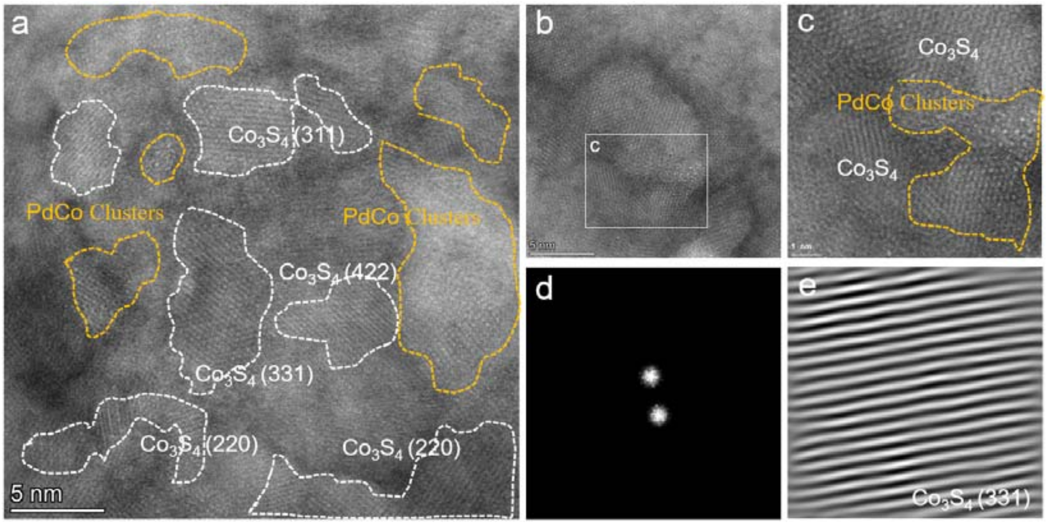

Figures S9. STEM images of PdCo-Co_3S_4 after the HER durability test.

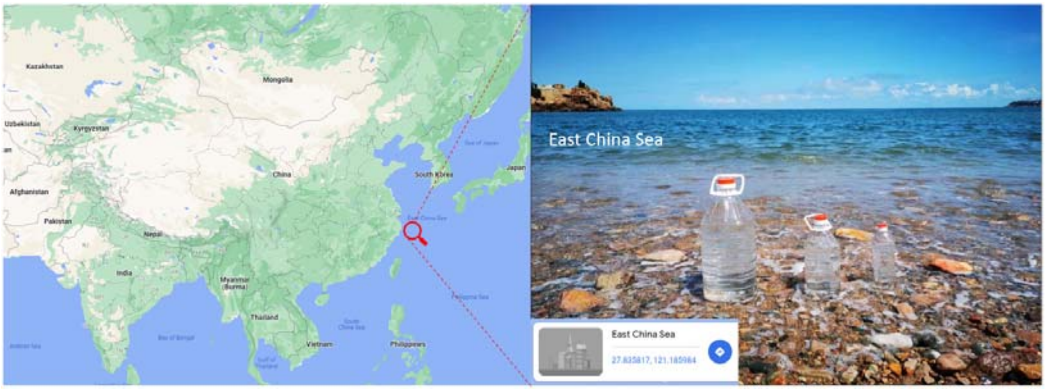

Figures S10. Natural seawater derives from the East China Sea.

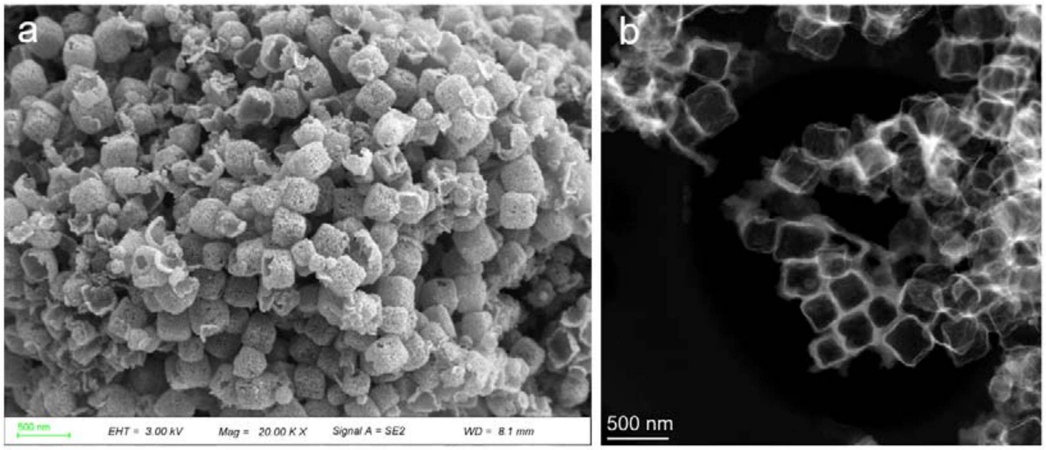

Figures S11. (a) SEM and (b) STEM images of PdCo-Co_3S_4 after the seawater splitting durability test.

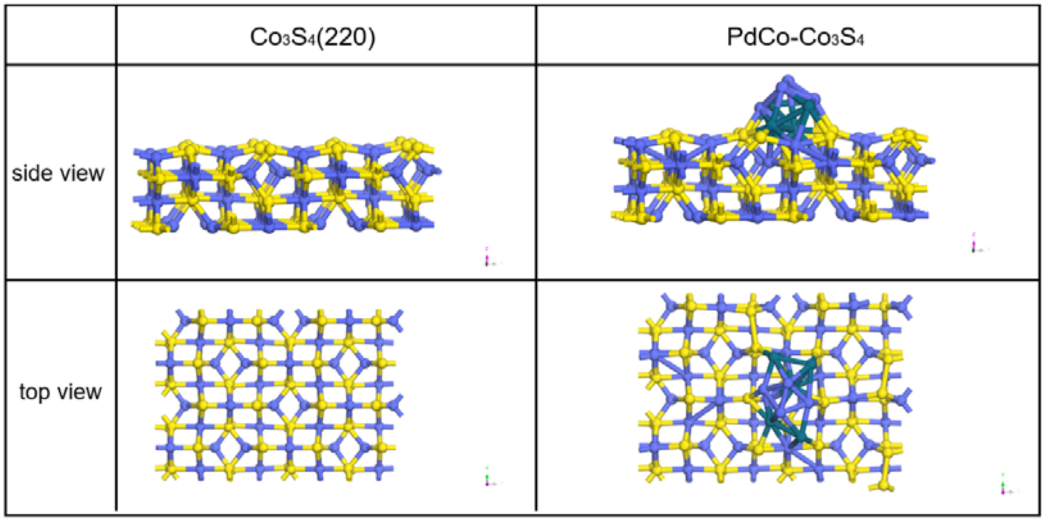

Figures S12. Theoretical model of the Co_3S_4(220), and PdCo-Co_3S_4 after optimization.

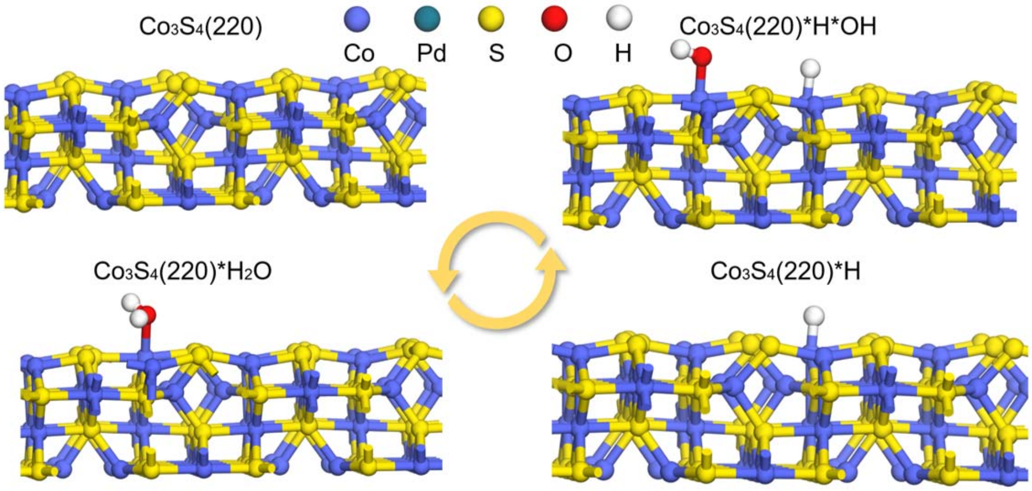

Figures S13. The atomic model of Co_3S_4 and the proposed HER pathway.

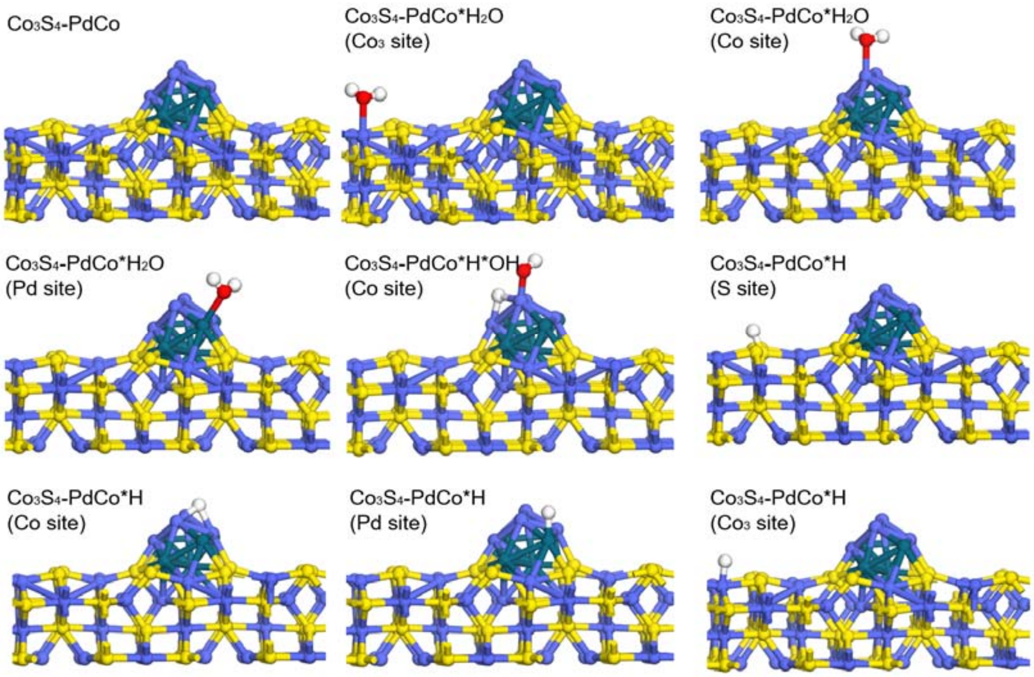

Figures S14. The atomic model of PdCo-Co_3S_4 and the proposed HER pathway on Co, Pd, S and Co_3 activity sites.

Supporting Tables

Table S1. EXAFS fitting parameters at the Co K-edge for various samples（S_0^2=0.73 from Co-foil）

	**shell**	**CN**[a]	**R**[b]**(Å)**	**σ^{2c}(Å^2)**	**ΔE_0**[d]**(eV)**	**R factor**
Co foil	Co-Co	12	2.49±0.01	0.0063	7.1±0.4	0.0013
PdCo-Co_3S_4	Co-S	3.7±0.3	2.13±0.01	0.0029	-12.4±3.0	0.0101

[a]*CN*: coordination numbers; [b]*R*: bond distance; [c]σ^2: Debye-Waller factors; [d] ΔE_0: the inner potential correction. R factor: goodness of fit. Error bounds that characterize the structural parameters obtained by EXAFS spectroscopy were estimated as CN±20%; R ± 1%; σ^2 ± 20%.

Table S2. HER performances of $PdCo\text{-}Co_3S_4$ and other reported electrocatalysts in the literature.

**Catalyst**	**Electrolyte**	η_{10}**(mV)** (*j*=10mA cm^{-2})	**Tafel slope** (mV dec^{-1})	**Reference**
$PdCo\text{-}Co_3S_4$	1 M KOH	96	22.23	**This work**
Co_3O_4/Co_3S_4@NF	1 M KOH	134	99	1
Co_3S_4@NFs	1 M KOH	161.2	126.96	2
$Ni\text{-}Co_3S_4$	1 M KOH	262	116.3	3
$CoS_{1.097}/MoS_2$	1 M KOH	249	1.16	4
$Co\text{-}1T\text{-}MoS_2$	1 M KOH	240	82	5
$NiMo_3S_4$	1 M KOH	257	98	6
MoS_2/MoO_2	1 M KOH	240	76.1	7
$T\text{-}MoS_2$	1 M KOH	290	120	8
CoO_x@CN	1 M KOH	232	115	9
MoC_x	1 M KOH	151	59	10
Mo_2C	1 M KOH	270	78	11
$NiCo_2S_4/Co_9S_8$	1 M KOH	172	115	12
$Co_9S_8\text{-}CoSe_2$	1 M KOH	150	89	13
$CoMoO_4/Co_9S_8$	1 M KOH	172	78	14
$Co_xP/Co_9S_8\text{-}Cu$	1 M KOH	118	67	15
Co_9S_8 / MoS_x	1 M KOH	161	78	16
$Mn\text{-}N\text{-}Co_9S_8$	1 M KOH	102	107	17
TiO_2/Co_9S_8	1 M KOH	150	71	18
HfCoS/rGO	1 M KOH	164	49	19
Fe_7S_8/CoS	1 M KOH	216	117	20

Supporting References

[1] Wang Q, Xu H, Qian X, et al. Oxygen and sulfur dual vacancy engineering on a 3D Co_3O_4/Co_3S_4 heterostructure to improve overall water splitting activity. Green Chemistry, 2022, 24(23): 9220-9232.

[2] Peng O, Shi R, Wang J, et al. Hierarchical heterostructured nickle foam–supported Co_3S_4 nanorod arrays embellished with edge-exposed MoS_2 nanoflakes for enhanced alkaline hydrogen evolution reaction. Materials Today Energy, 2020, 18: 100513.

[3] Ji K, Che Q, Yue Y, et al. Ni-Doped Co_3S_4 Hollow Nanobox for the Hydrogen Evolution Reaction. ACS Applied Nano Materials, 2022, 5(7): 9901-9909.

[4] Sun J, Huang Z, Huang T, et al. Defect-rich porous $CoS_{1.097}/MoS_2$ hybrid microspheres as electrocatalysts for pH-universal hydrogen evolution. ACS Applied Energy Materials, 2019, 2(10): 7504-7511.

[5] Ma F, Liang Y, Zhou P, et al. One-step synthesis of Co-doped 1T-MoS_2 nanosheets with efficient and stable HER activity in alkaline solutions. Materials Chemistry and Physics, 2020, 244: 122642.

[6] J. Jiang, M. Gao, W. Sheng, Y. Yan, Hollow Chevrel-Phase $NiMo_3S_4$ for Hydrogen Evolution in Alkaline Electrolytes, Angew. Chem. Int. Ed. 55 (2016), 15240-15245.

[7] L. Yang, W. Zhou, D. Hou, K. Zhou, G. Li, Z. Tang, L. Li, S. Chen, Porous metallic MoO_2-supported MoS_2 nanosheets for enhanced electrocatalytic activity in the hydrogen evolution reaction, Nanoscale 7 (2015), 5203-5208.

[8] Y. Liu, J. Wu, K.P. Hackenberg, J. Zhang, Y.M. Wang, Y. Yang, K. Keyshar, J. Gu, T. Ogitsu, R. Vajtai, J. Lou, P.M. Ajayan, Brandon C. Wood, B.I. Yakobson, Self-optimizing, highly

surface-active layered metal dichalcogenide catalysts for hydrogen evolution, Nature Energy 2 (2017).

[9] Jin H, Wang J, Su D, et al. In situ cobalt–cobalt oxide/N-doped carbon hybrids as superior bifunctional electrocatalysts for hydrogen and oxygen evolution. Journal of the American Chemical Society, 2015, 137(7): 2688-2694.

[10] Wu H B, Xia B Y, Yu L, et al. Porous molybdenum carbide nano-octahedrons synthesized via confined carburization in metal-organic frameworks for efficient hydrogen production. Nature communications, 2015, 6(1): 6512.

[11] Morales-Guio C G, Thorwarth K, Niesen B, et al. Solar hydrogen production by amorphous silicon photocathodes coated with a magnetron sputter deposited Mo_2C catalyst. Journal of the American Chemical Society, 2015, 137(22): 7035-7038.

[12] Basu M. Nanotubes of $NiCo_2S_4/Co_9S_8$ heterostructure: efficient hydrogen evolution catalyst in alkaline medium. Chemistry–An Asian Journal, 2018, 13(21): 3204-3211.

[13] Chakrabartty S, Karmakar S, Raj C R. An electrocatalytically active nanoflake-like Co_9S_8-$CoSe_2$ heterostructure for overall water splitting. ACS Applied Nano Materials, 2020, 3(11): 11326-11334.

[14] Du X, Huang C, Zhang X. Synthesis of $CoMoO_4/Co_9S_8$ network arrays on nickel foam as efficient urea oxidation and hydrogen evolution catalyst. International Journal of Hydrogen Energy, 2019, 44(36): 19595-19602.

[15] Jiang D, Ma W, Zhou Y, et al. Coupling Co_2P and CoP nanoparticles with copper ions incorporated Co_9S_8 nanowire arrays for synergistically boosting hydrogen evolution reaction electrocatalysis. Journal of colloid and interface science, 2019, 550: 10-16.

[16] Herbaut M, Siaj M, Claverie J P. Nanomaterials-based water splitting: how far are we from a sustainable solution?. ACS Applied Nano Materials, 2021, 4(2): 907-910.

[17] Xing Y, Li D, Li L, et al. Accelerating water dissociation kinetic in Co_9S_8 electrocatalyst by mn/N Co-doping toward efficient alkaline hydrogen evolution. International Journal of Hydrogen Energy, 2021, 46(11): 7989-8001.

[18] Yang F, Deng S, Lin S, et al. Porous TiO_2/Co_9S_8 core-branch nanosheet arrays with high electrocatalytic activity for a hydrogen evolution reaction. Nanotechnology, 2019, 30(40): 404001.

[19] Itagi, Mahesh, Deepak Chauhan, and Young-Ho Ahn. HfCoS/rGO Bifunctional Electrocatalysts for Efficient Water Splitting in Alkaline Media. Energy & Fuels (2023).

[20] Xu Y, Feng T, Wang Y, et al. Constructing bifunctional Fe_7S_8/CoS heterostructures for efficient water electrolysis. International Journal of Hydrogen Energy, 2023, 48(1): 113-122.

[21] Kresse, G.; Furthmuller, J. Efficient Iterative Schemes for ab initio Total-energy Calculations Using a Plane-wave Basis Set. Phys. Rev. B 1996, 54, 11169.

[22] Perdew, J. P.; Burke, K.; Ernzerhof, M. Generalized Gradient Approximation Made Simple. Phys. Rev. Lett. 1996, 77, 3865.

[23] Hammer, B.; Hansen, L. B.; Norskov, J. K. Improved Adsorption Energetics within Density-functional Theory Using Revised Perdew-Burke-Ernzerhof Functionals. Phys. Rev. B 1999, 59, 7413.

[24] Grimme, S. Semiempirical GGA-type Density Functional Constructed with a Long-range Dispersion Correction. J. Comput. Chem. 2006, 27, 1787-1799.
